# Asthma-Targeted MURs: How Confident are Community Pharmacists in Delivering Different Interventions?

**DOI:** 10.3390/pharmacy7030079

**Published:** 2019-07-01

**Authors:** Reem Kayyali, Ifrah Ali, Asma’a Al-Hindawi, Iman Hesso, Finlay Royle

**Affiliations:** 1 Pharmacy and Chemistry, School of Life Sciences, Kingston University, Kingston upon Thames KT1 2EE, UK; 2 NHS Lambeth Clinical Commissioning Group, Lambeth SE1 7NT, London, UK

**Keywords:** community pharmacists, confidence, asthma, respiratory patients, medicine use reviews

## Abstract

This study aimed to identify and determine the confidence level of community pharmacists in providing different interventions during asthma-targeted medicine use reviews (MURs). A self-administered questionnaire was posted to 487 pharmacies accredited to provide the service, across Greater London, Southampton, Cornwall, Sheffield and Norwich. A total of 122 responses were obtained, giving a response rate of 25% (122/487). Around half of the community pharmacists (51.6%) were providing more than 60 asthma-targeted MURs annually with inhaler technique being the most offered intervention and stepping up/down therapy being the least. The majority of community pharmacists (94.3%) were confident in providing inhaler technique advice, followed by smoking cessation (91%). However, confidence was less with relevant vaccination (61.5%) and stepping up/down patients’ therapy (56.6%). Confidence level can vary between community pharmacists regarding different interventions provided during respiratory MURs. The results stress the need to promote community pharmacists’ confidence in providing interventions such as stepping up/down therapy during asthma-targeted MURs. Additional research in this field is highly recommended in order to evaluate community pharmacists’ confidence level on a national scale and to determine the factors influencing it. The study also suggests that provision of different interventions during respiratory MURs can be related to how community pharmacists perceive their role.

## 1. Introduction

Asthma is a chronic respiratory condition characterised by airflow limitation that can be reversed. Symptoms include: Wheezing, cough, breathlessness and tightness in the chest. In England, it is estimated that 5.4 million patients suffer from asthma, making England the country with the highest prevalence of asthma in the world. In addition, 75% of asthma related hospital admissions are avoidable and as many as 90% of the deaths from asthma are preventable [1]. Evidence of the positive impact pharmacists can have in optimising the management of respiratory patients in the community is well established in the literature [2,3,4]. In England, medicine optimisation is a key target for the National Health Service (NHS) [5]. Respiratory patients, including asthma patients, can be managed by community pharmacists through the Medicine Use Review (MUR) service which was introduced in 2005 in England to promote medicine optimisation for patients with long-term conditions [5,6]. An MUR involves a patient-pharmacist consultation that is offered annually to discuss the patient’s understanding and use of medications. The service has been introduced with the aim of improving patient satisfaction with medication related information and adherence, hence improving patient outcomes and reducing medication wastage. Target patients for this service include: patients taking high-risk medications, patients with respiratory conditions, patients recently discharged from hospital and patients at risk of or diagnosed with cardiovascular disease and regularly being prescribed at least 4 medications. Pharmacists can also provide patients with healthy living advice including smoking cessation, physical activity, vaccination (mainly flu vaccination), and nutrition during an MUR consultation. The NHS remunerates a fee of £28 per MUR. Pharmacists may communicate the MUR results to the patient’s general practitioner by sending an MUR feedback report [5,6,7,8]. The NHS has also introduced the seasonal influenza vaccination service in community pharmacies since July 2015 with respiratory patients being a target group [9].

Despite the availability of the MUR service for over a decade, there is relatively little evidence to support its general effectiveness in England [4]. However, respiratory MURs have been suggested to be the most beneficial in England [8,10]. Additionally, a recent randomised controlled trial (RCT) in Italy provided a robust evidence base behind the effectiveness of respiratory MURs in terms of asthma control and medication adherence compared to usual care [4]. Interestingly, in a recent qualitative research conducted by the authors in England, the MUR service was identified by community pharmacists to be the main service to support regular respiratory patients [11].

Therefore, this study aimed to identify and determine the confidence level of community pharmacists in providing different interventions during asthma-targeted MURs in England. 

## 2. Materials and Methods

### 2.1. Research Design

A quantitative approach was used to address the aim of this study. A short paper-based, self-administered questionnaire was used as a data collection tool. The questionnaire was designed by the researchers to address the aim of this study, and was divided into 4 sections: number of MURs conducted, interventions done during respiratory MURs and pharmacists’ confidence with respect to these interventions, training and resources, and demographics (Supplementary S1). The questionnaire consisted of 13 questions, consisting predominantly of closed-ended questions of different styles (multiple choice, yes/no questions and rating questions). The invitation was addressed to the responsible community pharmacist in the pharmacy including those working as manager, full time, part-time or on a locum basis. The questionnaire was circulated via postal services to community pharmacies across Sheffield, Norwich, Southampton and Cornwall and four boroughs in Greater London (Kingston upon Thames, Richmond upon Thames, Wandsworth and Lambeth). The community pharmacists were informed about the rationale and the nature of the study via a cover letter which was distributed with every questionnaire. The questionnaire, accompanied with a pre-paid envelope for return, was sent to independent, supermarket, small chain and large chain community pharmacies that were accredited to provide the MUR service. The list of pharmacies in each area was identified via the NHS choices website. The total number of community pharmacies in the included areas in the study is 487 (32 Kingston, 63 Wandsworth, 67 Lambeth, 45 Richmond, 71 Cornwall, 44 Southampton, 37 Norwich, 128 Sheffield) [12,13,14,15]. Sample size calculation was performed using the Raosoft sample size calculator [16], indicating a sample size of 215 at a 95% confidence level and 5% margin of error.

Community pharmacists were given a deadline of two months to send the completed questionnaire. A follow-up questionnaire pack was sent as a reminder after one month. Responses were received between January and March 2015. Only one response was obtained from each pharmacy. Only fully completed questionnaires were considered for analysis.

### 2.2. Pilot Study

A pilot study was conducted after obtaining the ethical approval with 5 pharmacies in East London to test the survey for content and face validity. Content validation focused on asking the pharmacists to complete the survey to verify whether suitable findings could be deduced from the questionnaire outcomes. Face validation incorporated asking pharmacists about the clarity and ease of completion of the questions. Only minor amendments were required. To avoid bias, results of the pilot study were excluded.

### 2.3. Data Analysis

Data entry and descriptive statistics were performed by three researchers using Microsoft Excel and SPSS version 24 software. A chi square test was performed to evaluate whether demographics impacted the number of MURs conducted yearly by the community pharmacists. Additionally, another chi square test was performed to evaluate the differences in perceived confidence level in relation to the number of MURs performed. To facilitate the analysis and reporting of the results of the Likert-scale questions related to confidence level, the options ‘very confident’ and ‘confident’ as well as ‘very unconfident’ and ‘unconfident’ were respectively clustered each into one group. The level of statistical significance was set at p < 0.05.

### 2.4. Ethical Consideration

Ethical approval for this study was obtained by the Delegated Research Ethics Committee at Kingston University London (Reference No. 1213/045).

## 3. Results

### 3.1. Response Rate and Demographics

487 questionnaires were distributed across Greater London, Sheffield, Norwich, Southampton and Cornwall. A total of 122 usable questionnaires were completed and returned for analysis, giving an overall response rate of 25%. The demographics of the participants are summarised in Table 1.

### 3.2. Provision of Asthma Respiratory MURs and Resources

Around half of the pharmacists (51.6%, n = 63/122) were providing more than 60 asthma MURs per year, followed by 29 pharmacists (23.8%) providing between 41 and 60 MURs, 17 (13.9%) providing between 21 and 40 MURs and only 13 (10.7%) providing less than 20 asthma MURs per year. Most respondents (68%, n = 83/122) reported to spend an average of 10-20 minutes in conducting asthma MURs and nearly a third (29.5%, n = 36/122) reported to spend less than 10 minutes. Interestingly, no statistically significant impact was observed with respect to demographic characteristics such as age (p = 0.753), gender (p = 0.808) and years of experience (p = 0.287) on the number of MURs conducted annually by the respondents.

Most participants (69.7%, n = 85/122) received specific training for conducting respiratory MURs. However, responses were mixed when pharmacists were asked if they perceive there is a need for further training to improve the provision of respiratory MURs, with 46.7% (n = 57/122) indicating the need for further training and 53.3% (n = 65/122) indicating no need. Community pharmacists were also required to indicate what resources could improve the quality of asthma MURs. In this regard, 44.3% (n = 54/122) of community pharmacists chose structured checklists, followed by 38.5% (n = 47/122) for training and 36.1% (n = 44/122) for clinical guidelines. The majority of community pharmacists (n = 98/122, 80.3%) also provided additional suggestions to improve the quality of asthma MURs including: additional time (n = 66), better communication with general practitioners (n = 32) and having standard operating procedures (n = 21).

### 3.3. Interventions Provided During Asthma-Targeted MURs and Confidence Level

The vast majority of community pharmacists (n = 118/122, 96.7%) reported to provide inhaler technique advice to patients during asthma-targeted MURs, followed by smoking cessation (n = 74/122, 60.7%). However, less than a third of community pharmacists (n = 36/122, 29.5%) reported to provide relevant vaccinations (e.g., Flu vaccination) and only 20.5% (n = 25/122) reported to step up/down therapy for patients during asthma MURs.

Community pharmacists were required to rank their confidence level in the different interventions done during asthma-targeted MURs. The vast majority were very confident/confident in providing inhaler technique and smoking cessation, whereas two-third were very confident/confident in providing relevant vaccination and more than half were very confident/confident in stepping up/down therapy for patients. Nearly one-third (31.1%) of the community pharmacists could not clearly determine/rank their confidence level with respect to stepping up/down therapy and chose the neutral category. As for provision of relevant vaccination, equal numbers of community pharmacists were either neutral or very unconfident/unconfident. Results are summarised in Table 2.

Furthermore, a chi square test was performed to examine potential differences in participants’ confidence level based on the number of MURs conducted annually. There was a statistically significant difference in confidence level pertaining to inhaler technique advice (p = 0.037) in relation to the number of MURs conducted annually by the respondents. However, the results did not achieve statistical significance with respect to confidence levels related to smoking cessation (p = 0.732), flu vaccination (p = 0.387) and stepping up/down therapy (p = 0.796).

## 4. Discussion

Despite that all community pharmacists in this study provided asthma-targeted MURs, confidence in providing different interventions during these MURs varied considerably. Confidence has been reported to be among the barriers that affect the pharmacists’ provision of extended services [17] such as the MUR service [10,18], and other services as well [19]. Factors influencing confidence levels were beyond the scope of this study. However, the reported need for additional training by around half of the respondents in the current study may provide a valid explanation behind the reported confidence levels, given that training was reported to affect services provision in the literature [17,19,20,21]. In a previous research in England, community pharmacists reported lack of confidence in providing lifestyle advice to patients with cardiovascular diseases due to lack of proper training and regular practice [19]. This stresses the importance of promoting confidence among community pharmacists in all interventions that can be done during respiratory MURs, given that the MUR service was found to be the main service to support respiratory patients [11] and the positive potential these MURs can have on the management of respiratory patients [4,10]. In one study in England, asthma-targeted MURs were reported to be beneficial to patients in terms of inhalers checking, and confidence and understanding about their treatment and condition [10]. The latest evidence in this domain is the RCT conducted by Manfrin et al. [4] in Italy. The latter study demonstrated how the provision of asthma-targeted MURs by community pharmacists was beneficial to patients’ asthma control and cost-effective compared to usual care [4].

On the other hand, the current results may be related to community pharmacists’ perceptions of their role rather than confidence. In the current study, the vast majority of community pharmacists reported to provide inhaler technique advice as opposed to stepping up/down therapy. A previous qualitative research in England highlighted community pharmacists’ lack of recognition of a clinical role while supporting respiratory patients [11]. This could provide a plausible explanation for the limited stepping up/down therapy performed during an MUR, considering that more than half of the community pharmacists in the current study reported to be confident in that regard.

This study should be viewed in the light of certain limitations including the low response rate and the fact that confidence level was self-reported (subjective) by community pharmacists, which affects the generalisability of the reported results. Furthermore, flu vaccination is now offered as an advanced service in community pharmacies since July 2015, hence confidence in provision of vaccination among community pharmacists during respiratory MURs should have changed with an expected increase compared to the results of the current study being conducted between January and March 2015. Given that the current study is limited to a low response rate, a similar study on a national scale and with a higher response rate would be recommended to establish a more profound insight into the status quo, given the increased recognition of the impact community pharmacists can have in the management of patients with chronic conditions, including respiratory patients. Additionally, the current research highlights the need for further research into the factors that would influence confidence levels in this domain.

## 5. Conclusions

The current study suggests that community pharmacists are less confident in providing interventions such as stepping up/down patients’ therapy and relevant vaccination during asthma-targeted MURs and stresses the need to promote confidence in these areas. Additional research in this domain is highly recommended in order to evaluate the confidence level of community pharmacists on a national scale and to determine factors influencing their confidence. The study also suggests that provision of different interventions during respiratory MURs can be related to how community pharmacists perceive their role.

## Figures and Tables

**Table 1:** Demographics of the participating community pharmacists.

Participants Characteristics	Number (n)	Percentage (%)
**Gender**		
Male	69	56.6
Female	53	43.4
**Age**		
< 25 y	9	7.4
25–35 y	54	44.3
36–45 y	26	21.3
46–55 y	19	15.6
> 55 y	14	11.5
**Years of Experience as Community Pharmacist**		
0–5 y	44	36.1
6–10 y	26	21.3
11–15 y	11	9
> 16 y	41	33.6
**Location**		
Greater London	56	45.9
Norwich	19	15.6
Southampton	24	19.7
Sheffield	15	12.3
Cornwall	8	6.6
**Job Type**		
Full time	49	40.2
Part time	4	3.3
Locum	20	16.4
Superintendent	9	7.4
Owner	8	6.6
Manager	32	26.2
**Pharmacy Type**		
Independent	68	55.7
Small chain (<20 pharmacies)	12	9.8
Large chain (>20 pharmacies)	40	32.8
Supermarket	2	1.6
**Total N= 122**		

**Table 2:** Confidence level in different interventions provided during asthma MURs.

Intervention		Confidence Level	
	Very confident /Confident, n (%)	Neither confident nor unconfident, n (%)	Very unconfident /Unconfident, n (%)
Inhaler technique	115 (94.3)	5 (4.1)	2 (1.6)
Smoking cessation	111 (91)	7 (5.7)	4 (3.3)
Relevant vaccination (e.g., Flu vaccination)	75 (61.5)	24 (19.7)	23 (18.8)
Stepping up/down therapy	69 (56.6)	38 (31.1)	15 (12.3)

## Supplementary Materials

The following are available online at https://www.mdpi.com/2226-4787/7/3/79/s1, Materials S1: A survey to evaluate the optimisation of asthma therapy in community pharmacy.
