# Supplementary material for: Asthma-Targeted MURs: How Confident are Community Pharmacists in Delivering Different Interventions?
Source: Pharmacy (Basel). 2019 Jul 1;7(3):79. doi: 10.3390/pharmacy7030079 (PMC6789459; doi:10.3390/pharmacy7030079)
Supplement: Supplementary file 1 [file pharmacy-07-00079-s001.pdf]

pharmacy

A survey to evaluate the optimisation of asthma therapy in community pharmacy

Number of MURs conducted

1. How many respiratory MURs do you conduct in a year?

0-20 ☐ 21-40 ☐ 41-60 ☐ 60+ ☐

2. On average, how long do you spend conducting an MUR?

<10 mins ☐ 10-20 mins ☐ 21-30 mins ☐ >30 mins ☐

Training provided to conduct respiratory MURs

3. Have you received any further training, which may help you conduct an asthma MUR?

   Yes ☐ No ☐

4. Do you feel more training needs to be provided?

   Yes ☐ No ☐

5. What in your opinion could improve the quality of asthma MURs? (Select as many as appropriate)

☐ Structured checklist
☐ Training
☐ Clinical guidelines
☐ Other, please specify below

Interventions conducted in asthma-targeted MURs and pharmacist's confidence

6. What are the main interventions that you make as a result of an asthma MUR? (Select as many as appropriate)

☐ Inhaler technique
☐ Smoking cessation
☐ Relevant vaccinations (Flu and Pneumococcal vaccination)
☐ Stepping up/down therapy
☐ Other, please specify below

----

7. How confident are you in making the following recommendations as a result of an asthma MUR?

	Very unconfident	unconfident	Neither confident, nor unconfident	Confident	Very confident
Inhaler technique					
Smoking cessation					
Relevant vaccinations (e.g.: flu)					
Stepping up/down therapy					

Demographics

8. What is your gender?

Male ☐ Female ☐

9. How old are you?

Under 25 ☐ 25-35 ☐ 36-45 ☐ 46-55 ☐ Over 55 ☐

10. How many years of experience as a community pharmacist do you have?

0-5 years ☐ 6-10 years ☐ 11-15 years ☐ 16-20 years ☐ > 20 years ☐

11. What type of pharmacy do you work in?

☐ Independent
☐ Small chain pharmacy (20 pharmacies or less)
☐ Large chain pharmacy (more than 20 pharmacies)

12. What position do you hold in the pharmacy?

- [ ] Owner
- [ ] Superintendent
- [ ] Pharmacy manager
- [ ] Pharmacist
- [ ] Locum

13. Any additional comments

You have reached the end of the survey. Thank you for taking time to complete.
